# Retinal Microvascular Changes in Internal Carotid Artery Stenosis

**DOI:** 10.3390/jcm12186014

**Published:** 2023-09-16

**Authors:** Bilge Batu Oto, Oğuzhan Kılıçarslan, Yasemin Kayadibi, Aslıhan Yılmaz Çebi, İbrahim Adaletli, Senihe Rengin Yıldırım

**Affiliations:** 1 Department of Ophthalmology, Cerrahpaşa Medical Faculty, Istanbul University-Cerrahpaşa, 345098 Istanbul, Turkey; 2 Department of Ophthalmology, Ayancık State Hospital, 57400 Sinop, Turkey; 3 Department of Radiology, Cerrahpaşa Medical Faculty, Istanbul University-Cerrahpasa, 345098 Istanbul, Turkey; 4 Department of Ophthalmology, Çerkezköy State Hospital, 59500 Tekirdağ, Turkey

**Keywords:** internal carotid artery stenosis, OCTA, retinal vasculature, superficial capillary plexus, deep capillary plexus, foveal avascular zone

## Abstract

**Purpose:** We aimed to analyze retinal microvascular parameters, measured by optical coherence tomography angiography in patients with internal carotid artery stenosis compared to healthy individuals. **Materials and Methods:** A total of 41 eyes from 30 patients who had varying degrees of carotid stenosis, and 42 eyes from 42 healthy controls, were enrolled in this study. Depending on the degree of stenosis evaluated by Doppler ultrasonographic imaging, the patient group was further subclassified into mild, moderate, and severe carotid artery stenosis. Superficial and deep capillary plexus vessel densities, radial peripapillary capillary vessel density, foveal avascular zone, and flow densities in the choriocapillaris and outer retina were evaluated by optical coherence tomography angiography. **Results:** The superficial and deep capillary plexus vessel densities were significantly reduced among the groups, only sparing the foveal region. The mean superficial plexus vessel density was 45.67 ± 4.65 and 50.09 ± 4.05 for the patient and control group, respectively (*p* = 0.000). The mean deep capillary plexus density was 46.33% ± 7.31% and 53.27% ± 6.31% for the patient and control group, respectively (*p* = 0.000). The mean superficial and deep capillary vessel densities in the foveal region did not show any statistical difference between the patient and control groups (*p* = 0.333 for the superficial and *p* = 0.195 for the deep plexus vessel density). Radial peripapillary capillary vessel density was decreased in the patient group (*p* = 0.004). The foveal avascular zone area was wider in the patient group but this difference did not show a significant difference (*p* = 0.385). **Conclusions:** Retinal microvascular changes are a prominent outcome of internal carotid disease, and even mild stenosis can lead to alterations in the retinal microvascular bed which could be detected by OCTA. By early detection of microvascular changes in the retina in this patient group, we might speculate the overall vascular condition.

## 1. Introduction

Internal carotid artery stenosis (CAS) directly affects the ocular blood supply and may cause ocular ischemic syndrome, which has a mortality rate of 40% within 5 years of onset [1]. CAS is a prominent risk factor for ophthalmic ischemic complications such as amaurosis fugax, central retinal artery occlusion, and ocular ischemic syndrome since the ophthalmic artery is a branch of internal carotid artery (ICA) and the central retinal artery arises from the ophthalmic artery. Transient monocular visual loss that can be induced by exercise, position, and bright light amaurosis is another typical clinical phenomenon associated with CAS [2]. An intramural hematoma may cause stenosis and eventual trombus formation leading to carotid dissection as a severe complication. Ophthalmic manifestations of CAS can arise from emboli of atheroma plaque, direct hypoperfusion of ocular vasculature by ICA stenosis, or via collaterals formed after ICA stenosis [3]. Especially retinal arterial occlusion with multiple emboli can be associated with severe carotid occlusive disease [4]. Lawrence et al. showed that the incidence of ocular symptoms increased in CAS cases with occlusion greater than 50% [5]. Therefore, early detection of any impairment in ocular blood flow and mild retinal ischemia is essential to prevent ocular ischemic complications.

Grading of carotid obstruction is crucial for the management and follow-up of CAS. Especially, those with more than 50% carotid stenosis are considered hemodynamically significant. Doppler ultrasound is a valuable technique to make this classification.

Different medical or surgical treatments are performed to prevent the complications of CAS. Carotid artery stenting and balloon angioplasty or carotid endarterectomy are mainly used. The North America Symptomatic Carotid Endarterectomy Trial (NASCET) suggested that patients with more than 70% occlusion benefited most from carotid endarterectomy [6]. In a prospective study, carotid endarterectomy was found to increase the peak systolic blood flow of the ophthalmic artery by Doppler ultrasound after one week of surgery in patients with chronic ocular ischemic syndrome [7].

Ocular findings are not always very deterministic for identifying CAS. Before fundus findings and visual symptoms, imaging of retinal vascular structure may be an earlier marker for CAS. Visual symptoms may not become evident until severe stenosis is fully established, causing optic nerve dysfunction. Optical coherence tomography angiography (OCTA) is a novel, dye-free, non-invasive retinal imaging technique that allows the quantitative analysis of retinal vascular parameters. OCTA may reveal microvascular abnormalities before they become clinically apparent. In cardiovascular diseases, macular and peripapillary vessel density reduction has previously been reported [8]. It is well known that optic nerve head vascular insufficiency may lead to ganglion cell loss, and optic nerve vascular supply is affected in severe carotid stenosis. Though severe stenosis may lead to clinical signs and symptoms, patients with mild stenosis are usually asymptomatic. But vascular insufficiency, both in the optic nerve head and macula, may still lead to ultrastructural changes. With OCTA, these changes could be subjectively detected.

In this current study, we aimed to analyze retinal microvascular parameters by OCTA in patients with CAS compared to healthy individuals.

## 2. Materials and Methods

### 2.1. Study Population

This cross-sectional study includes patients who had internal carotid stenosis, and healthy controls. Typical signs and symptoms like amaurosis fugax, transient visual loss, and transient ischemic attack were primary indications for carotid artery imaging. Patients with those symptoms and other asymptomatic patients with specific indications who had undergone Doppler ultrasonography in the Radiology Department of İstanbul University Cerrahpasa—Cerrahpasa Medical Faculty were evaluated. All imaging was performed by the same radiologist (YK). The degree of stenosis was evaluated by the grading method suggested by the Society of Radiologists at the Ultrasound Consensus Conference. According to this Doppler, US patients were sub-classified into the categories of normal (no stenosis), mild (<50% stenosis), moderate (50–69% stenosis), and severe (≥70% stenosis to near occlusion) [9]. Patients with any degree of stenosis and age- and gender-matched healthy controls were enrolled in the study. OCTA measurements were taken from ipsilateral eyes of carotid stenosis in patients with unilateral disease, and bilateral eyes were scanned in patients with bilateral stenosis. The control group had also undergone Doppler ultrasound imaging to confirm the absence of stenosis.

Patients with major ocular or systemic diseases were excluded from the study. Eyes with CAS-related complications such as ocular ischemic syndrome, central/branch retinal artery occlusion, anterior ischemic optic neuropathy, and macular diseases like age-related macular degeneration, vitreomacular interface disorders, macular telangiectasia, as well as patients with diabetic retinopathy and history of vitreoretinal surgery, were excluded. Eyes with optic neuritis/neuropathies, intraocular hypertension or glaucoma, and orbital mass were excluded. Refractive errors between ± 3D spherical equivalents were included in the study.

Patients with systemic diseases, namely heart failure, aortic stenosis or aneurysm, any other obstruction of the proximal vascular system from ICA, neurodegenerative diseases, any intracranial mass lesions, cerebrovascular accidents rather than transient ischemic attacks, hematologic diseases and malignancies were excluded.

The study was conducted according to the tenets of the Declaration of Helsinki. Informed consent was obtained from patients. The study was approved by the ethics committee of Istanbul University-Cerrahpasa, Cerrahpasa Medical Faculty Ethics Committee for Registering Clinical Trials (07/04/2021-70066).

### 2.2. Examination and OCTA Analysis

All patients underwent comprehensive ophthalmologic examination. Accompanying diseases, medications, and histories of surgeries were noted. Best corrected visual acuity was measured with the Snellen chart. Slit lamp biomicroscopy and dilated fundus examination were performed. Pupillary dilatation was provided via one drop of 1% cyclopentolate and 1% tropicamide. Intraocular pressure was measured via non-contact tonometry before pupillary dilatation. After a complete examination, Spectral Domain OCTA scans were obtained from patients and controls with RTVue XR Avanti (Optovue, Inc. Fremont, CA, USA). Under pupillary dilatation, 6 × 6 mm HD Angio Retina and 4.5 × 4.5 mm HD Angio Disc measurements were performed for each individual. Automatic segmentation of the retinal layers was applied to derive the en face slabs for each retinal capillary plexus. The superficial capillary plexus (SCP) was defined from the inner limiting membrane (ILM) to 9 μm above the inner plexiform layer (IPL), the deep capillary plexus (DCP) was defined from 9 μm above the IPL to 9 μm below the outer plexiform layer (OPL), and the choriocapillaris layer was defined from 9 μm above and 30 μm below the Bruch membrane layer. Macular vessel densities were calculated according to Early Treatment of Diabetic Retinopathy Study (ETDRS) chart of macula. The device creates 3 circles with a diameter of 1 mm-3 mm-6 mm, respectively, starting from the center of the fovea. Area of the most inner circle at 1 mm diameter was accepted as fovea. Annulus between 1 mm diameter and 3 mm diameter circle was accepted as parafovea. Annulus between 3 mm diameter and 6 mm diameter circle was accepted as perifovea. Vessel density was calculated as the proportion of the measured area occupied by blood vessels. We analyzed vessel density for fovea, parafovea, and perifovea regions. Superficial capillary plexus vessel density (SCP-VD) and deep capillary plexus vessel density (DCP-VD) were measured in whole image, fovea, parafovea, and perifovea. Foveal avascular zone (FAZ) area was measured in the retina slab from ILM to 9 μm below the outer plexiform layer. Radial peripapillary capillary (RPC) density and the peripapillary thickness were automatically calculated by the software AngioVue 2.0.; and inside disc, peripapillary, and whole image vessel densities were recorded. Eye-tracking system was activated during the application to gain more accurate images and to minimize motion artifacts. Images with scan qualities below 7/10 were excluded for correct evaluation. Flow parameters were calculated in the outer retina and choriocapillaris layer. Flow areas were measured in these layers as an area that gave flow signals in a circular area with a one-millimeter radius centered in the fovea. A 0.75 mm-wide elliptical annulus delineated by automated fitting extending from the optic disc margin was utilized to measure RPC vessel density (RPC-VD) in the circumpapillary area.

### 2.3. Statistical Analysis

All analyses were performed using SPSS version 22.0 software (IBM Corp., Chicago, IL, USA). Quantitative data were expressed as the mean ± standard deviation (SD), median [interquartile range (IQR) and number (%)] depending on the type and distribution of the data. Normal distribution was examined using visual (histogram and Q-Q plot) and analytical methods (Shapiro–Wilk test). The demographic data of the patients with carotid stenosis and healthy controls were compared using the Pearson chi-squared test or Fisher exact test for categorical variables (e.g., sex) and Mann–Whitney U test for continuous variables (e.g., age). Statistical significance between the patient and healthy control groups was evaluated by independent *t*-test when the data showed normal distribution and Mann–Whitney U test when the data did not show normal distribution. Categorical variables were expressed as numbers and percentages and compared using the chi-squared test. Spearman rank correlation or Pearson correlation analysis were used to analyze the relationship between the degree of carotid stenosis and OCTA parameters.

## 3. Results

### 3.1. Study Population

A total of 41 eyes from 30 patients with varying degrees of carotid stenosis were enrolled in this study. The ipsilateral eyes of the carotid stenosis side were enrolled and the stenotic side was further subclassified into three groups depending on the degree of carotid stenosis. A total of 21 carotids had mild, 16 had moderate, and 4 had severe stenosis. The median age of the patient group was 63 (14) (range 32–87) years. The control group comprised 42 eyes from 42 age- and gender-matched healthy subjects. The demographics of the study group are given in Table 1. No relations between the age and SCP-VD and DCP-VD were observed in the study group. (Pearson correlation *p* = 0.005 for both SCP-VD and DCP-VD).

### 3.2. Comparison of OCTA Parameters in Patients with Carotid Stenosis and the Healthy Controls

Superficial capillary plexus vessel density in the patient group compared to healthy controls was lower in the foveal, perifoveal, and parafoveal region, with a marked statistical significance in the perifoveal and parafoveal regions, and whole image (Table 2).

**Figure 1:**
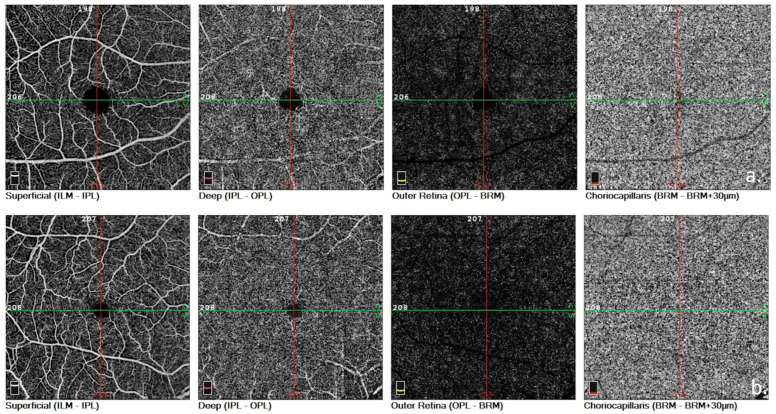
Comparison of superficial and deep vessel plexuses. (**a**) Optical coherence tomography angiography image of a patient with carotid stenosis, (**b**) Optical coherence tomography angiography image of a healthy control. Enlargement in FAZ could be observed in the upper image which belongs to a patient with carotid stenosis.

Deep capillary plexus vessel density was lower in the patient group compared to the healthy controls in all measured regions except the fovea. Density in the deep capillary plexus, parafoveal and perifoveal regions, and whole image was significantly reduced (Table 3). Figure 1 shows the comparison of SCP-VD and DCP-VD in a patient with severe stenosis and a healthy subject.

**Figure 2:**
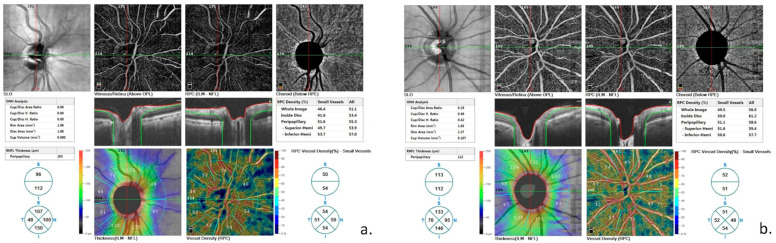
Radial peripapillary capillary density in a patient with carotid stenosis (**a**) and a healthy control (**b**).

Peripapillary RPC-VD was significantly lower in the CAS group than in healthy controls. Though statistically insignificant, RPC-VD whole density was also lower in the patient group compared to health controls (Figure 2). The FAZ area was wider in the patient group compared to healthy controls but this difference did not show statistical significance (Table 4). The foveal flow area of the choriocapillaris was the same between the groups, whereas it was significantly reduced in the outer retina in patients with CAS (Table 4).

**Figure 3:**
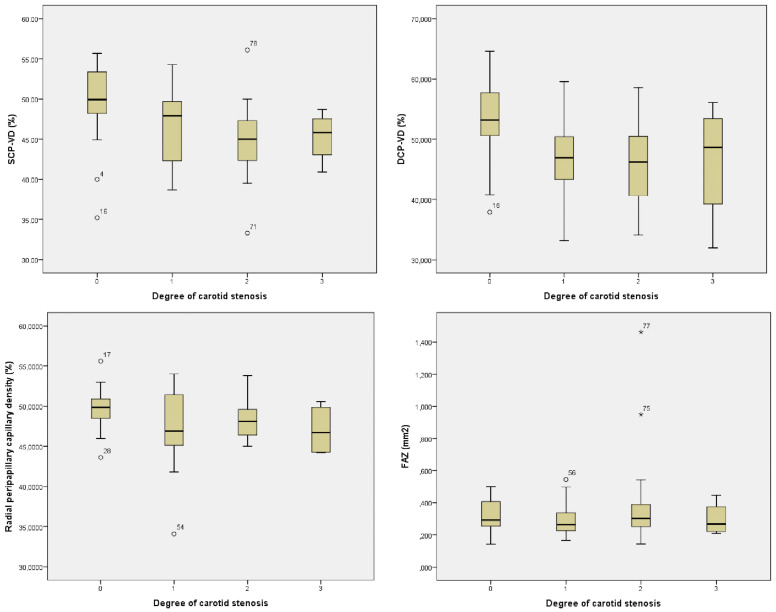
Boxplot graphic comparing superficial deep capillary plexus vessel density, radial peripapillary capillary density, and FAZ in healthy control and patients with different degrees of stenosis (0: no stenosis, 1: mild stenosis, 2: moderate stenosis, 3: severe stenosis).

Comparison of mild, moderate, and severe CAS did not show any differences in SCP-VD, VCP-VD, and FAZ (Table 5). Mean SCP-VD, DCP-VD, RPC-VD, and FAZ values in control, and in mild, moderate, and severe degrees of CAS are given in Figure 3. SCP-VD and DCP-VD were negatively correlated with the degree of stenosis (Spearman’s rho = −0.502, *p* = 0.000, and Spearman’s rho = −0.404, *p* = 0.000 for the SCP-VD and DCP-VD, respectively). A negative relation was observed between the RPC-VD and degree of stenosis, though statistically insignificant (Spearman’s rho = −0.298, *p* = 0.008).

## 4. Discussion

We aimed to demonstrate the retinal microvascular changes in patients with CAS and determine how much the degree of stenosis affects retinal microcirculation. It has been reported that even mild CAS is strongly related to ocular ischemic syndrome and stroke [10,11]. By early detection of microvascular changes in the retina in this patient group, we might speculate the overall vascular condition of patients and take timely measures to prevent further damage even before visual acuity is affected. Liu J. et al. showed that retinal microperfusion changes in the superficial vascular layer were correlated with hemodynamic alterations in the brains of patients with moderate or severe ICA stenosis, demonstrating that retinal perfusion might give insight into cerebral perfusion [12]. Stenosis of the internal carotid artery would result in decreased blood flow to the choriocapillaris and central retinal artery, causing apoptosis of retinal ganglion cells, cone cells, and rod cells. Given that vascular insufficiency impacts visual function, OCTA may be a valuable tool to detect early microvascular changes in the retina. The microvascular changes in the retina may also be used as an indicator of cardiovascular diseases [13,14].

Overall, SCP-VD and DCP-VD were significantly reduced with foveal sparing only. FAZ area did not show significant difference. RPC-VD in the whole image was also decreased in the patient group but there was no statistical significance. RPC-VD in the peripapillary region, though, was significantly reduced in the patient group.

Liu et al. showed that SCP-VD was lower in patients with carotid stenosis greater than 50%, compared to patients with mild stenosis with <50% stenosis and healthy controls [15]. Nevertheless, they found no difference in DCP-VD and FAZ areas in patients with severe stenosis, mild stenosis, and healthy controls. Lahme et al. also reported that a reduction in SCP-VD was more prominent than DCP-VD in patients with carotid stenosis greater than 70% [16]. A significant improvement in DCP-VD was reported in patients with CAS after carotid artery angioplasty and stenting [17], demonstrating that patients with severe CAS benefited from treatment. Microvascular improvements after treatments could be detected solely by OCTA.

In contrast to the previous literature, which reported an effect on the SCP-VD but not the DCP-VD [15,16,18], we observed that both the superficial and deep capillary plexus vessel densities showed statistically significant differences among groups, only sparing the foveal region. The choriocapillaris, superficial, and deep retinal layers are all supplied by the internal carotid artery. Therefore, we expect all layers to be affected in CAS, rather than only SCP-VD. Though vessel densities of superficial and deep capillary plexus in the fovea were lower in the patient group, this was not statistically significant. This result may demonstrate a foveal sparing even in marked vascular insufficiency. A possible autoregulation of foveal microvasculature as a physiologic protective mechanism remains to be highlighted. A previous study showed mean retinal sensitivity was lower in patients with moderate CAS compared to healthy controls, but retinal sensitivity in the foveal region did not differ between groups, and the authors concluded that the reason was possibly related to a larger number of cones in the foveal area [19]. Another study found that CAS mainly affects the rod-mediated response [20]. The foveal sparing observed in our study may also be due to this.

The RPC forms a unique plexus of a capillary network around the optic nerve head. A histological study revealed that the RPC has a distinct morphological appearance as long, straight capillaries parallel to the nerve fiber bundles with sparse anastomoses to other vessels [21]. Our study found that peripapillary RPC-VD was significantly lower in patients with CAS, indicating that the optic nerve head vascular supply is significantly reduced. This vascular insufficiency may further lead to apoptosis of retinal ganglion cells and result in functional loss of the optic nerve. Inside-disc RPC-VD had the same distribution among patients and healthy controls. The whole image RPC-VD was also significantly lower in the patient group, consistent with the previous literature [16,18].

The FAZ was wider in patients with CAS; but did not demonstrate a statistical significance. Some other studies also found wider FAZ [18]. The flow density was significantly reduced in patients with CAS. Likewise, some previous studies reported lower macular and optic nerve flow densities in patients with moderate and severe CAS [15]. To our knowledge, no studies have reported the flow area measurements in CAS patients. In our study, decreased flow area in the outer retina was prominent, whereas the flow area in the choriocapillaris was the same among groups. Analysis related to the choriocapillaris may be challenging and require accurate segmentation and quantification [22]. The measurements related to the choriocapillaris may therefore constitute a nonsignificant difference in our study.

In our study, we observed that SCP-VD and DCP-VD were negatively correlated with the degree of stenosis. Both SCP-VD and DCP-VD decreased as the degree of stenosis worsened. In addition, FAZ area and RPC-VD were lessened as the degree of stenosis increased but this relation was statistically insignificant. More accurate analysis could be made in patient groups in which degree of stenosis is evenly distributed.

Our study comes with a few limitations. First, the small sample size and unequal distribution of patients in each group prevented the multivariate analysis. The effect of the degree of carotid stenosis on retinal microvasculature could not be evaluated efficiently. The primary reason for the result that various degrees of stenosis did not change the OCTA parameters is thought to be insufficient and unequal distribution in each group. Secondly, no retinal functional tests which may reveal a link between anatomic alterations and retinal functions were performed. Most patients were using multiple medications, namely statins, antihypertensives, and anticoagulants. No analyses were made regarding the medications used by the patients, which could have a potential effect on OCTA.

In conclusion, we observed a significant reduction in SCP-VD and DCP-VD with foveal region sparing. The foveal sparing could be attributed to a protective mechanism of cones. Flow area and peripapillary RPC-VD were also affected in the patient group. Intergroup analysis of patients did not reveal any significant differences in the abovementioned parameters. No correlation between the disease severity and retinal microvascular insufficiency could be made, possibly due to the unequal distribution in each group. Retinal microvascular changes are a prominent outcome of internal carotid disease, and even mild stenosis can lead to alterations in the retinal microvascular bed which could be detected by OCTA. Since the majority of patients in our patient group consisted of patients with mild stenosis, we can conclude that OCTA could be used to recognize mild disease. OCTA may be used as a tool for monitoring the microvascular changes associated with carotid stenosis and give an insight concerning the overall vascular condition of the patient, since OCTA gives subjective data concerning vessel density and flow.

## Figures and Tables

**Table 1:** Demographics of patients and control group.

	Patient Group (n:30)	Control Group (n:42)	*p*
**Gender (female/male)**	13/17	18/24	0.968 ^α^
**Median age [IQR]** **(years)**	63 (14) (range 32–87)	60 (7) (range 50–75)	0.123 ^β^

n: number, IQR: interquartile range, ^α^ Mann–Whitney U test, ^β^ Chi-square test.

**Table 2:** Superficial capillary plexus vessel density in patient and control groups.

	Patient Group	Control Group	*p*
**SCP-VD %** **Whole image**	45.67 ± 4.65	50.09 ± 4.05	0.000 ^α^
**SCP-VD %** **Fovea**	16.63 ± 5.96	17.92 ± 6.05	0.333 ^β^
**SCP-VD %** **Parafovea**	47.34 ± 5.75	52.44 ± 4.77	0.000 ^α^
**SCP-VD %** **Perifovea**	46.39 ± 4.99	50.66 ± 4.08	0.000 ^α^

SCP-VD: superficial capillary plexus vessel density, ^α^: Mann–Whitney U test, ^β^: independent *t*-test.

**Table 3:** Deep capillary plexus vessel density in patient and control groups.

	Patient Group	Control Group	*p*
**DCP-VD %** **Whole image**	46.33 ± 7.31	53.27 ± 6.31	0.000 ^β^
**DCP-VD %** **Fovea**	32.88 ± 9.53	35.18 ± 6.95	0.195 ^β^
**DCP-VD %** **Parafovea**	52.49 ± 5.63	56.78 ± 4.12	0.000 ^β^
**DCP-VD %** **Perifovea**	46.98 ± 7.79	55.10 ± 6.83	0.000 ^β^

DCP-VD: deep capillary plexus vessel density, ^β^: independent *t*-test.

**Table 4:** FAZ, Flow area, and RPC vessel density in patient and control groups.

	Patient Group	Control Group	*p*
**FAZ area (mm^2^)**	0.344 ± 0.230	0.324 ± 0.097	0.385 ^α^
**Flow area (mm^2^)** **Outer Retina**	0.476 ± 0.370	0.953 ± 0.487	0.000 ^α^
**Flow area (mm^2^)** **Choriocapillaris**	2.069 ± 0.142	2.105 ± 0.070	0.121 ^β^
**RPC-VD whole density %**	47.65 ± 3.87	49.66 ± 2.13	0.006 ^β^
**RPC-VD peripapillary %**	50.11 ± 4.31	52.48 ± 2.62	0.004 ^α^
**RPC-VD inside disc %**	48.73 ± 5.1	49.48 ± 4.76	0.502 ^β^

RPC-VD: Radial peripapillary capillary vessel density, FAZ: foveal avascular zone, ^α^: Mann–Whitney U test, ^β^: independent *t*-test.

**Table 5:** Superficial, deep capillary plexus vessel density, FAZ, and Flow area of various degrees of stenosis groups.

	Mild Stenosis	Moderate Stenosis	Severe Stenosis	p^1^ ^(Mild–Moderate Stenosis)^	p^2^ ^(Mild–Severe Stenosis)^	p^3^ ^(Moderate–Severe Stenosis)^
**SCP-VD (%)**	46.54 ± 4.66	45.0 ± 4.90	45.3 ± 3.2	0.650	0.896	0.692
**DCP-VD (%)**	53.5 ± 6.2	47.1 ± 7.4	46.3 ± 10.3	0.370	0.539	0.946
**FAZ (mm^2^)**	0.29 ± 0.1	0.41 ± 0.33	0.29 ± 0.1	0.572	0.572	0.572

SCP-VD: superficial capillary plexus vessel density, DCP-VD: deep capillary plexus vessel density, FAZ: foveal avascular zone, P^1^: mild-moderate stenosis, P^2^: mild-severe stenosis, P^3^: moderate-severe stenosis. Independent samples Kruskal–Wallis test was performed in analysis between groups.

## Data Availability

Research data could be shared upon request to the corresponding author.
